# Mouse Models and Tools for the *in vivo* Study of Neutrophils

**DOI:** 10.3389/fimmu.2019.03130

**Published:** 2020-01-21

**Authors:** Julien Stackowicz, Friederike Jönsson, Laurent L. Reber

**Affiliations:** ^1^Institut Pasteur, Department of Immunology, Unit of Antibodies in Therapy and Pathology, UMR INSERM 1222, Paris, France; ^2^Sorbonne Université, Collège Doctoral, Paris, France; ^3^Center for Pathophysiology Toulouse-Purpan (CPTP), UMR 1043, University of Toulouse, INSERM, CNRS, Toulouse, France

**Keywords:** neutrophils, Cre-recombinase, diphtheria toxin, mouse models, depletion, Gfi-1, NETosis

## Abstract

Neutrophils are the most abundant leukocytes in human blood and critical actors of the immune system. Many neutrophil functions and facets of their activity *in vivo* were revealed by studying genetically modified mice or by tracking fluorescent neutrophils in animals using imaging approaches. Assessing the roles of neutrophils can be challenging, especially when exact molecular pathways are questioned or disease states are interrogated that alter normal neutrophil homeostasis. This review discusses the main *in vivo* models for the study of neutrophils, their advantages and limitations. The side-by-side comparison underlines the necessity to carefully choose the right model(s) to answer a given scientific question, and exhibit caveats that need to be taken into account when designing experimental procedures. Collectively, this review suggests that at least two models should be employed to legitimately conclude on neutrophil functions.

## Introduction

Neutrophils are key players of the immune system. They are the first leukocytes to be recruited to inflammatory sites (1) and they can contribute to inflammation through several mechanisms. These include their capacity to engulf and eliminate pathogenic agents or particles through phagocytosis, an NADPH oxygenase-dependent mechanism with reactive oxygen species synthesis and antibacterial enzymes mobilization (2). Moreover, neutrophils can also release lipid mediators, such as PAF or LTB4, thereby facilitating the recruitment of circulating cells (3). Furthermore, neutrophils can release Neutrophil Extracellular Traps (NETs), which are composed of DNA decorated with proteins such as cathepsins, histones, neutrophil elastase and myeloperoxidase (MPO) (4). NETs are sticky weblike structures, which are thought to trap pathogens and toxins, thereby inactivating them and preventing them from spreading (4, 5).

Mouse models of human diseases have progressively been developed in parallel with techniques and strategies to deplete neutrophils, to knockout genes for key neutrophil enzymes, and to visualize these cells *in vivo*. Traditional models for the study of neutrophils, including models of depletion or mutant mice, allowed a global comprehension of neutrophil biology. Nevertheless, these models notably raised questions of specificity, and hence justified the generation of novel models in recent years (6, 7). Together, these advances rendered mouse models advantageous for the study neutrophils in health and disease. Here, we summarize and discuss currently available mouse models for neutrophil depletion and highlight the main models deficient in key neutrophil enzymes. Moreover, strategies to visualize neutrophils and NETs *in vivo* are examined.

## Inducible Depletion of Neutrophils

One commonly used approach to study the role of a given cell type is to deplete the cell type of interest *in vivo* in order to characterize the resulting phenotype. Numerous studies have used inducible neutrophil depletion strategies, as they enable to control neutrophil deficiency at different stages of interest. Furthermore, drugs and depleting antibodies can be used in virtually all mouse strains, and are thus convenient and versatile tools for the study of neutrophil biology.

### Cyclophosphamide

Cyclophosphamide is a pro-drug that is used in humans as an antitumor agent (8). The designation “prodrug” is due to the fact that cyclophosphamide needs to be metabolized by liver enzymes such as cytochrome P450 for the formation of alkylating cytotoxic agents (9). Metabolized cyclophosphamide triggers the formation of DNA crosslinks and lesions (9) that lead to cell cycle arrest and cell death, thereby limiting the proliferation of dividing cells (10). This explains its use as an antitumor drug. Treatment of mice with cyclophosphamide increases the susceptibility of mice to pathogenic agents and has been used for the development of mouse models of infection (11, 12). Indeed, intraperitoneal injection of cyclophosphamide triggers the death of hematopoietic stem cells and incapacitates remaining cells preventing their proliferation and differentiation (13). Neutrophils are rather short-lived cells (14, 15). Hence, pharmacological depletion of hematopoietic stem cells is associated with an almost complete disappearance of blood neutrophils as early as 3–4 days after intraperitoneal or subcutaneous cyclophosphamide injection (11, 16).

This background explains the choice of this drug to assess the role of neutrophils *in vivo* (17–19). Mice are usually treated with a high dose of cyclophosphamide (150 mg/kg) on day 0 and with a low dose (100 mg/kg) 3 days later (11, 16, 17, 20). Three to four days after the last treatment with cyclophosphamide, mice exhibit a strong neutrophilia with a 3-fold increase of blood neutrophils compared to untreated mice (11, 16). Indeed, repetitive injections are necessary if long-term effects of neutrophil depletion are to be evaluated (17). A clear advantage of the use of cyclophosphamide to induce neutropenia in mice is the relatively low price of this drug, and its capacity to render any mouse strain neutropenic. However, a major limitation of this approach is the fact that cyclophosphamide is all but neutrophil specific. Indeed, cyclophosphamide-treated mice also exhibit markedly reduced numbers of circulating monocytes, B and T cells (11, 16, 17, 20). These confounding factors render the interpretation of results obtained in cyclophosphamide-treated animals challenging. For example, after treatment with cyclophosphamide, *Streptococcus pneumoniae*-infected mice showed a higher number of lung colony-forming units (CFUs) (21). However, the role of neutrophils in controlling lung CFU numbers has since come into question. Marks et al. treated *S. pneumoniae*-infected mice with neutrophil depleting antibodies and revealed they had similar CFUs to mice treated with an isotype control antibody, a difference that was explained by the lack of specificity of cyclophosphamide (22). In conclusion, while cyclophosphamide represents a convenient first tool to predict the role of neutrophils *in vivo*, findings obtained using this drug require confirmation with other approaches to address neutrophil biology.

### Vinblastine

Vinblastine is another cytostatic drug (23) that can bind to tubulin molecules, thereby disrupting the assembly of microtubules (24) and interfering with cytoskeletal dynamics. Thus, vinblastine targets dividing cells and leads to mitotic arrest (25). Treatment of mice with vinblastine induces strong neutropenia (26, 27). Nevertheless, this drug is rarely used to study neutrophils *in vivo*. Overall, vinblastine exhibits the same disadvantages as cyclophosphamide. It displays a poor selectivity in blood (as illustrated by a respective 35 and 39% reduction in blood monocytes and lymphocytes) (26), and induces cytotoxicity in other cell types such as pancreatic cells (28) and during spermatogenesis (29). Furthermore, vinblastine might affect any cellular process involving microtubule assembly, limiting its use for *in vivo* experiments.

### Depleting Antibodies

Neutrophil depletion can also be induced by the systemic administration of specific antibodies. As pharmacological drugs, depleting antibodies are efficient in WT mice and most knockout mice, which circumvents the necessity to generate mutant mice.

#### Anti-Gr-1

The monoclonal rat IgG2b antibody RB6-8C5 was originally reported to specifically bind to neutrophils (30, 31), and recognize the surface molecule Gr-1. Treatment of mice with RB6-8C5 anti-Gr-1 antibodies leads to a profound neutropenia (32–34) that lasts for up to 3–5 days depending on the injected dose (32, 35). Early reports suggested that RB6-8C5-mediated depletion was neutrophil-specific and would not affect other cell types such as monocytes (31, 33). These findings were however challenged by the findings that mice infected with the helminth *Nippostrongylus brasiliensis* also exhibited a severe reduction of blood eosinophils upon RB6-8C5 injection (36), and that RB6-8C5 treatment could induce a decrease of blood and spleen monocytes and memory-type CD8^+^ T cells (35, 37). A more precise analysis of Gr-1 revealed that Gr-1 represents a family of two GPI-anchored proteins, Ly6C, and Ly6G (30). Ly6G is specifically expressed on the surface of mouse neutrophils (30), and thus represents a good candidate to selectively target neutrophils and trigger their depletion *in vivo*. Ly6C, however, is mainly expressed on neutrophils and monocytes but also in macrophages, and subpopulations of CD8^+^ T cells (38–41), which could account for the lack of selectivity of RB6-8C5 antibodies.

Neutrophil depletion by RB6-8C5 was reported to be unaffected by the treatment of mice with Fc-block (anti-FcγRII/III antibodies) or in FcRγ-knockout mice (34). Disruption of complement activation by pre-treatment with cobra venom factor delayed—but did not prevent—the RB6-8C5-induced neutrophil depletion (34), suggesting that neutrophil depletion occurs through complement-dependent and -independent pathways. The role of complement is not likely to be dependent on the membrane attack complex since neutrophils are not sensitive to membrane attack complex-induced death (42). However, C3-deficient mice were shown to exert no depletion upon RB6-8C5 treatment (43), indicating that opsonization of neutrophils is a prerequisite for their depletion. Moreover, sterile inflammation in the peritoneum can be induced by injection of thioglycolate triggering a peritonitis with a characteristic sequential change in cell population and cell maturation. Following activation of receptor for Advanced Glycation Endproducts (44, 45), neutrophils are the first cells to be recruited to the injection sites followed by monocytes, which become the main cell population between day 3 and 6 of peritonitis. In this context, pre-treatment of mice with RB6-8C5 antibodies induced neutrophil apoptosis, suggesting that other pathways could participate in neutrophil depletion in thioglycolate-induced inflammatory conditions (46). Injection of RB6-8C5 antibody in TNF-α pre-treated mice induces death due to microvessel obstruction and coagulation resulting in respiratory defects (34, 47). This is thought to be a consequence of neutrophil activation following binding of RB6-8C5 antibodies to TNF-α-primed cells (34, 47). These observations further reveal that RB6-8C5-induced depletion cannot be used in every context due to potential adverse reactions triggered by the antibody itself (as observed in TNF-α-primed mice). Other limitations of RB6-8C5-induced depletion include a side-specific depletion pattern that needs to be considered when planning experiments. Indeed RB6-8C5 was reported to be inefficient in the liver (48). Finally, RB6-8C5 treatment may trigger other side effects, such as myeloid cell expansion and upregulation of macrophage markers, probably through STAT-1, 3, and 5 engagement (46).

Despite the now well-described lack of specificity, RB6-8C5 antibodies have been used extensively (alone or in combination with other approaches) to study the role of neutrophils in various disease models, including models of infection (35, 49, 50), arthritis (51, 52), wound repair (53) and anaphylaxis (54).

#### Anti-Ly6G

The rat IgG2a antibody 1A8 has been described as being specific for Ly6G (30), and therefore as interacting specifically with neutrophils (55). Intraperitoneal injection of 1A8 induces a nearly complete depletion of blood and spleen neutrophils (35, 55), and an 80% decrease of liver neutrophils (35), while monocyte counts remain unaffected (35, 55). However, 1A8 is less efficient than RB6-8C5 antibody for the induction of neutrophil depletion. Indeed, mice are usually treated with a 2-fold higher dose of 1A8 than RB6-8C5 (55, 56). Furthermore, 2 days post-treatment, neutrophil counts rise again in 1A8-treated mice while it is not the case in RB6-8C5-treated mice (55). Finally, 1A8 efficiency has recently been questioned, as 1A8-treated 24 weeks old C57BL/6J mice showed no neutrophil depletion (57). The same observation was conducted with bone marrow neutrophils from BALB/c mice (58). To circumvent this low efficacy, it was recently suggested that combining 1A8 treatment with a secondary antibody injection could lead to a 90% depletion of blood neutrophils (57). Nevertheless, this statement needs to be tempered since the 1A8-induced neutrophil depletion was still efficient in 9 and 24 weeks old BALB/c and FVB/N mice, and in 9 weeks old C57BL/6J mice (57). Furthermore, 1A8-induced neutrophil depletion was also questioned in PbNK65-infected C57BL/6 mice and CFA-challenged BALB/c mice (58) and further demonstrates the need to verify the depletion efficacy when using this antibody.

Studies investigating the *in vivo* mechanism underlying 1A8-induced depletion described that depletion of macrophages prior to 1A8-treatment decreases the efficiency of neutrophil depletion (59), suggesting that macrophages are key effector cells for neutrophil depletion (59). This was confirmed by intravital microscopy, revealing that neutrophils opsonized with fluorescently labeled 1A8 antibody were phagocytized by macrophages in the spleen, liver and bone marrow (60). Interestingly, the same group reported that the choice of fluorochrome had an influence on the depletion efficiency and suggested that this might be due to differences in the binding of the labeled antibodies to Ly6G. For instance, 1A8-FITC was more efficient at inducing neutrophil depletion than 1A8-APC (60).

1A8 antibody has been extensively used to study the contribution of neutrophils *in vivo*. It was for example used to show that neutrophils support metastasis formation (61), limit muscle alteration after intense exercise (62), and participate in protection against *S. pneumoniae* (63). Moreover, with the availability of this more neutrophil-specific antibody, several studies reassessed the role of neutrophils *in vivo*. For instance, in a context of Herpes Simplex Virus type-1 infection, treatment of mice with RB6-8C5 had resulted in an increased sensitivity of the mice toward the virus, suggesting a protective role of neutrophils (64, 65). However, the use of 1A8 antibody to deplete neutrophils did not alter the virus replication, questioning the previously described role of neutrophils in anti-viral reactions (65). Similar results were obtained for bacterial infections with *Listeria monocytogenes*. It was previously shown that RB6-8C5-treated mice failed to control *L. monocytogenes* infection and it was concluded that neutrophils played an important role in defense against this pathogen (49). More recently, it was verified that RB6-8C5-treated mice did not control *L. monocytogenes* infection. However, going further, it was also demonstrated that 1A8-treated mice showed the same *L. monocytogenes*-induced mortality as WT mice (66), questioning the requirement of neutrophils in this context. Using a model for monocyte specific depletion, it was demonstrated that monocyte-depleted mice died of infection (66), suggesting that monocytes are the key players in defense against *L. monocytogenes* whereas neutrophils are dispensable. This suggested that the phenotype of RB6-8C5-treated mice was due to the depletion of monocytes and not neutrophils.

NIMP-R14 is a second monoclonal antibody reported to recognize Ly6G (67). NIMP-R14 treatment leads to >95% depletion of spleen and blood neutrophils (20, 68–70). Neutrophil counts return to basal levels about 3 days post-NIMP-R14 injection (69), suggesting that repetitive injections are needed for long-term experiments (which is also the case for RB6-8C5 and 1A8). NIMP-R14 antibody has been used to study neutrophil functions in many disease models, including glomerulonephritis (71), anti-*Helicobacter pylori* immunity (72), autoimmune encephalomyelitis (73), and experimental models of anaphylaxis (68, 70). In contrast to 1A8 antibody driven depletion for which no unwanted side-effects have been described, several reports described a 2-fold decrease in blood monocytes and Ly6C^high^ spleen monocytes after NIMP-R14 treatment (68, 70, 74), questioning the specificity of this antibody.

While high doses of anti-Gr-1 and anti-Ly6G antibodies induce neutrophil depletion, these antibodies have also been used at a low dose to bind neutrophils without inducing cell depletion. This “low dose” strategy was used as a mean to assess the *in vivo* role of Ly6G. Injection of 10 μg of RB6-8C5 anti-Gr-1 or 5 μg of 1A8 anti-Ly6G antibodies prevented neutrophils from migrating toward inflamed tissues in a model of arthritis, suggesting that Ly6G is a key regulator of neutrophil migration (75). These findings were, however, challenged by the observation that RB6-8C5 anti-Gr-1 antibody-covered neutrophils show normal recruitment to the tissues in a mouse model *of Staphylococcus aureus* cellulitis (76). Moreover, Ly6G knockout mice (called Catchup mice) also showed normal neutrophil migration and functions in multiple models of sterile or infections inflammation (77). The binding of anti-Gr-1 and anti-Ly6G antibodies to neutrophils in the absence of neutrophil depletion highlights the necessity to appropriately evaluate the efficacy of their depletion. This is particularly critical in an inflammatory context, in which large numbers of neutrophils are released from the bone marrow. For instance, the TLR4 agonist carrageenan has extensively been used to induce acute inflammatory reactions, leading to the recruitment of neutrophils to the injection site (78). It has been shown that pre-treating mice with RB6-5C8 was sufficient to reduce the number of peritoneal neutrophils to 99%, even if mice were later intraperitoneally injected with carrageenan, 36 h after RB6-5C8 treatment (79). Furthermore, it is to note that neutrophils might be covered by anti-Gr-1 or anti-Ly6G antibodies, and thus appear as Ly6G negative cells in flow cytometric analysis. For example, in mice treated with anti-Ly6G NIMP-R14, bone marrow or lung neutrophils were not fully depleted but appeared as Ly6G-negative cells in flow cytometry analysis, due to epitope masking by NIMP-R14 antibodies (58, 70). As neutrophils are the major side scatter-high (SSC^high^) leukocyte population, we recommend to verify that treatment with a neutrophil-depleting antibody induces marked reduction in the number of SSC^high^ cells. Ideally, such analysis should be performed in combination with the use of complementary neutrophil markers to ascertain neutrophil depletion. These complementary markers include CD11b, Ly6C, or the 7/4-antigen, independently of the fact that these molecules are also expressed by other cell types such as monocytes or macrophages (80, 81). One possible strategy to identify “Ly-6G masked” neutrophils is to use either of the additional markers and subsequently exclude non-neutrophils from the gate by co-staining with other monocyte or lineage markers such as CD115 or B220 and including information from the forward vs. side scatter analysis (82). Alternatively, as anti-Ly6G and anti-Gr-1 antibodies are rat monoclonal antibodies, depleting antibody-covered neutrophils could be detected by revelation of the depleting antibodies themselves using fluorescently-labeled anti-rat IgG. Indeed, two studies used an anti-rat IgG2b to evaluate RB6-8C5-induced neutrophil depletion (48, 83).

The pharmacological approaches to deplete neutrophils are presented in Table 1.

**Table 1:** Pharmacological approaches to deplete neutrophils.

	**Name**	**Targets**	**Main advantages**	**Main limitations**	**References**
Drugs	Cyclophos-phamide	DNA	▪ Rapid and efficient ▪ Depletion can be performed in any mouse strain	▪ Poor specificity ▪ Needs repetitive injections	(11, 16, 17)
	Vinblastine	Tubulin	▪ Rapid and efficient ▪ Depletion can be performed in any mouse strain	▪ Poor specificity ▪ Needs repetitive injections	(26, 27)
Depleting antibodies	RB6-8C5	Gr-1 (Ly6G; Ly6C)	▪ Rapid and efficient ▪ Depletion can be performed in most mouse strains (exceptions include Ly6G^−/−^ Catchup mice)	▪ Also affects other Gr-1 expressing cell types (monocytes, eosinophils, memory-type CD8^+^ T cells) ▪ Repetitive injections needed for long-term depletion	(32, 34)
	1A8	Ly6G	▪ High specificity ▪ Depletion can be performed in most mouse strains (exceptions include Ly6G^−/−^ Catchup mice)	▪ Less efficient than anti-Gr-1 ▪ Requires high dose of antibodies ▪ Repetitive injections needed for long-term depletion	(35, 55, 56)
	NIMP-R14	Ly6G	▪ Rapid and efficient ▪ Depletion can be performed in most mouse strains (exceptions include Ly6G^−/−^ Catchup mice)	▪ Also affects Ly6C^hi^ monocytes ▪ Repetitive injections needed for long-term depletion	(68, 69)

### PMN^DTR^ Mice

Diphtheria toxin (DT) is a protein composed of two subunits that is released by *Corynebacterium diphtheriae* during infection (84). The human membrane-anchored form of the heparin-binding EGF-like growth factor (HB-EGF precursor) acts as the DT receptor (DTR) (85). Mouse HB-EGF precursor does not bind efficiently to DT, due to several amino acid exchanges in the HB-EGF domain that is critical for DT-receptor interactions. As a consequence, mouse cells show significantly enhanced resistance to DT-induced death (86).

By introducing the simian DTR into specific mouse cells, it is possible to render such cell populations sensitive to DT, and induce depletion of these cells upon DT injection (87). Indeed, binding of subunit B to the diphtheria toxin receptor (DTR) on human or monkey target cells induces entry of DT into the cell through receptor-mediated endocytosis (88). Once in the prelysosomal vesicles, a drop of pH induces the penetration of a domain of the DT subunit A through the vesicle membrane, leading to the release of DT into the cytoplasm (89). There, the DT subunit A binds to elongation factor 2 and inhibits protein synthesis and consequently triggers apoptosis (90).

It has been postulated that one molecule of DT can kill a eukaryotic cell (91), rendering this model of depletion extremely efficient. This is generally achieved using the “Cre-Lox” system. First, a loxP-flanked STOP cassette was introduced in the DTR gene (92) and this construct was inserted into the mouse locus Gt(ROSA)26Sor. The Rosa 26 is constitutively active and ubiquitously expressed in mice, which accounts for its success to drive transgenes and reporter constructs (92, 93). In the presence of Cre-recombinase, the STOP cassette is removed, allowing expression of the DTR. In the following, these mice will be referred to as iDTR mice (92).

One caveat of this method is the requirement of a promoter that is specific for the cell type of interest, in order to restrict expression of DTR to that particular cell type. hMRP8-Cre-ires/GFP mice were used to assess the specificity of the human MRP8 (hMRP8) promoter (6, 94–97). The transgenic construct encompasses an internal ribosome entry sites (98) that allows the concomitant expression of both the recombinase Cre and GFP under control of the myeloid cell-specific human MRP8 (hMRP8) promoter (94, 99). Using these mice, it was shown that GFP expression in mainly found in neutrophils (6, 95–97). However, it is important to note that hMRP8 promoter activity was also reported in 10–20% into some monocyte/macrophages populations (6, 97). Residual promoter activity was also initially noticed in a portion of granulocyte-macrophage progenitors (GMPs) (95). However, these findings were not confirmed in a more recent study (6).

Based on these promising findings, iDTR mice were crossed with hMRP8-Cre mice to generate a new mouse model of inducible neutrophil depletion [hereafter called PMN^DTR^ mice (6)]. Indeed, these mice exhibit an almost complete neutrophil depletion in the blood, bone marrow and spleen 24 h after a single intraperitoneal injection of 500 ng DT (6, 20). These results were confirmed by another group, which showed that subcutaneous injection of DT (10 ng/g mouse body weight) for three consecutive days results in depletion of about 90% of neutrophils in the blood and lungs (100). DT-injection did not affect the counts of most of the leukocyte populations tested, such as blood and spleen eosinophils, basophils, B, and T cells (6). The same results are obtained with peritoneal lavage fluid macrophages, mast cells, dendritic cells, B and T cells, and bone marrow eosinophils, basophils, B cells and GMPs (6). Neutrophils started to re-appear in the blood 2 days after DT treatment, reaching normal levels at day 3 (6). This highlights the fact that repetitive DT injections are required for long-term neutrophil depletion using PMN^DTR^ mice. Importantly, neutrophil depletion in PMN^DTR^ mice was also accompanied by a reduction in blood Ly6C^low^ and Ly6C^high^ monocytes, and in spleen Ly6C^high^ monocytes (6), confirming that some hMRP8-driven expression occurs in the monocyte lineage. This will have to be taken into account when interpreting results obtained using PMN^DTR^ mice.

One major advantage of this model is the possibility to perform the adoptive transfer of purified WT or mutant neutrophils into DT-treated PMN^DTR^ mice. This is achievable due to the fact that non-DTR expressing neutrophils will not be depleted by the DT treatment. For instance, DT-treated PMN^DTR^ mice were shown to be more sensitive to LPS-induced endotoxemia. Whereas, engraftment of these mice with WT neutrophils restored their resistance (confirming the protective role of neutrophils in this setting), this was not the case when transferred neutrophils were MPO-deficient, suggesting that neutrophil-derived MPO is necessary for the resistance against LPS (6). Bowers et al. transplanted bone marrow cells from control (DTR^−^) or PMN^DTR^ mice into recipient irradiated mice. One week later, mice were treated daily with DT for 7 days (i.p.), which resulted in a complete depletion of the granulocyte population (identified as Gr1^+^CD115^−^ cells) in mice transplanted with bone marrow cells from PMN^DTR^ mice but not from DTR^−^ mice (101).

Besides using the hMRP8 promoter to control Cre-recombinase expression, the latter was also inserted in the neutrophil-specific ly6G locus (77). These mice were recently crossed with iDTR mice as a novel model for neutrophil depletion (57). Unfortunately, upon DT injection, no drop in blood neutrophil counts was reported (57).

## Mouse Models With Constitutive Neutropenia

The rapid turnover of neutrophils and the capacity of the bone marrow to quickly increase neutrophil output in response to inflammatory signals in a process called emergency granulopoiesis (102) limits the use of inducible neutrophil depletion strategies for the study of long-term and inflammatory processes. Indeed, this would require repeated injections of DT or neutrophil-depleting antibodies. For instance, this could lead to the formation of anti-DT antibodies, lowering the efficiency of the depletion, as reported in CD11c-DTR mice (103). Several mouse models with constitutive neutropenia have been described and could theoretically circumvent these issues. However, constitutive neutropenia renders mice more susceptible to infections, and each of the constitutive neutropenic models presents with its own limitations that will be discussed below.

### G-CSFR^−/−^ Mice

A first mouse model with constitutive neutropenia was generated and described by Liu et al. (104). It is caused by the deletion of the Granulocyte Colony-Stimulating Factor Receptor (G-CSFR) gene. G-CSFR^−/−^ mice exhibit 80 and 50% decreased counts of blood and bone marrow neutrophils, respectively, and residual bone marrow neutrophils are more prone to apoptosis (104). This model was used to show the pro-inflammatory role of neutrophils in collagen-induced and antibody-induced arthritis (105, 106), leading to the proposal of G-CSFR as a new target in human rheumatoid arthritis (106). Additionally, an immunosuppressive function of neutrophils was put forward using this model, characterized by the neutrophil-dependent regulation of antigen-presentation by dendritic cells *in vivo* (107). Recently G-CSFR^−/−^ mice were shown to exhibit abnormally high responses in distal lymph nodes upon immunization, suggesting that neutrophils play a role in the restriction of the immune response to draining lymph nodes after immunization (108). Overall, however, G-CSFR^−/−^ mice are rarely used, probably due to the already mentioned residual neutrophils (7, 104) and the anticipated side effects caused by G-CSFR deficiency. Indeed, G-CSFR plays a major role in endothelial cell regulation (109), in the induction of migration and proliferation (110), in bone regeneration through osteoblast regulation (111) and in sympathetic nerve neurons signaling (112). Although an effect of G-CSFR deletion on these processes has not been extensively described in the literature, G-CSFR^−/−^mice likely exhibit several abnormalities that extend beyond their neutropenia.

### Cxcr2^−/−^ Mice

Neutrophil release from the bone marrow is tightly regulated by CXC chemokine receptors, such as CXCR2 and CXCR4. Whereas, neutrophils are retained in the bone marrow through constitutive expression of CXCL12 by bone marrow stromal cells acting on CXCR4, CXCR2 stimulation by Glu-Leu-Arg (ELR)^+^ chemokines induces the mobilization of CXCR2^+^ neutrophils (113). Comprehensively, Cxcr2^−/−^ mice (114) display a partial neutropenia with 60% decreased neutrophil counts in the blood compared to WT mice (113). Interestingly, spleen neutrophil counts are not affected by the deletion, whereas mature neutrophils in the bone marrow are twice more numerous in Cxcr2^−/−^ mice than in WT mice (113). This accumulation is accompanied by a lack of mobilization of Cxcr2^−/−^ neutrophils to the blood upon G-CSF treatment (113). Opposing the action of CXCR2, Cxcr4^−/−^ mice and WT mice treated with the CXCR4 blocking agent AMD3100 exhibit elevated blood neutrophil counts (113, 115). These two models can thus be used in studies requiring mice presenting with neutrophilia in the blood, whereas Cxcr2^−/−^ mice can be used as a model for mild neutropenia. A reduced neutrophil recruitment in Cxcr2^−/−^ mice was for instance described in contexts of Dextran Sodium Sulfate-colitis-induced acute kidney injury (116), *Toxoplasma gondii* infection (117), autoantibody-mediated arthritis (118), or posttraumatic neutroinflammation (119). Nevertheless, this model is rarely used. This is likely due to the relatively high number of residual neutrophils found in Cxcr2^−/−^ mice. Furthermore, CXCR2 is also expressed by monocytes/macrophages, mast cells, endothelial and epithelial cells (120–122), and the CXCR2 deficiency might thus also modify the biology of these cell populations. For instance, Cxcr2^−/−^ mice were shown to exhibit exaggerated inflammatory response to cutaneous and peritoneal inflammatory stimuli (123). Using anti-Ly6G antibodies to deplete neutrophils, the authors suggested that the inflammatory phenotype observed in Cxcr2^−/−^ mice might be neutrophil-independent, but depends on accumulation of a non-neutrophilic CXCR2 positive leukocyte population (123).

### Gfi-1-Deficient Mice

In 2002, a new mouse model was generated, in which the Gfi-1 (Growth factor independence-1) gene was inactivated through the removal of the exons 2 to 4 and partial removal of exon 5 (124). Gfi-1 had been described 9 years earlier, as encoding a protein that, if activated by a promoter insertion of proviruses could abolish the dependency of thymoma cells toward IL-2 (125). Through the analyses of the Gfi-1 gene sequence, it was predicted that the encoded protein could bind DNA through the engagement of zinc finger domains, and suggested that this interaction could lead to a down-regulation of the transcription of specific genes (125), as verified 3 years later (126). Before the generation of Gfi-1^−/−^ mice, Gfi-1 was therefore mainly investigated for its role in T cell differentiation and activation as well as in lymphomagenesis (125–128), thus restricting its spectrum of action to lymphoid cells.

However, following a more careful analysis of the Gfi-1 expression, it was first detected in the bone marrow (129) before it was demonstrated that its expression was not at all restricted to the lymphoid compartment. Indeed, granulocytes and LPS-activated macrophages also express the Gfi-1 gene (124).

One year after the first description of a Gfi-1^−/−^ mouse (124), a second strain was generated, in which the exons 1, 2, and a part of 3 were deleted, resulting again in mice unable to express Gfi-1 (130). Not surprisingly, both Gfi-1^−/−^ models develop a similar phenotype, with high mortality rates, reduced growth and decreased cell numbers in the thymus (124, 130). One of the most striking features of Gfi-1^−/−^ mice is the quasi absence of mature neutrophil numbers in the blood, spleen and bone marrow (124, 130), highlighting the role of this gene in the differentiation and/or survival of neutrophils. Furthermore, neutropenia is accompanied by the appearance of an abnormal cell population, described as “blastoid monocytic cells” or “atypical myeloid cells.” This population exhibits specific characteristics of the granulocyte lineage as well as of the macrophage lineage (130), such as ring-shaped nuclei and low expression levels of Gr-1 and Mac-1 together with high expression of Mac-3 and M-CSFR (124, 130). Interestingly, these cells are still capable of phagocytosis and oxidative burst (124, 130). Altogether, these characteristics indicate that the atypical myeloid cells, which develop in Gfi-1^−/−^ mice, share features of immature neutrophils and macrophage precursors (130).

Adding to the two Gfi-1^−/−^ mouse strains, another model of Gfi-1 deficiency was developed using the Cre-Lox system (131). Exons 4 and 5 of the Gfi-1 gene were flanked by two *LoxP* sites and Gfi-1^fl/fl^ mice were crossed with EIIa-Cre transgenic mice, to induce a constitutive deletion of Gfi-1 from the early embryo stage (132). Consistently with the two previous models, these Gfi-1^−/−^ mice exhibit abnormal features such as small body size and a high percentage of atypical myeloid cells (131). Using the CD4-promoter, this model enabled the restriction of the mutation to CD4-T cells, and thus, the assessment of the specific function of Gfi-1 in T-cells (131, 133).

Finally, a knock-in mouse model was generated in which the Gfi-1 gene was replaced by the gene encoding GFP (which they called Gfi-1^GFP/GFP^ mice) (134). This strain enabled the study of the expression and roles of Gfi-1 during T and B-cell differentiation and activation. Overall, Gfi-1^GFP/GFP^ mice display the same phenotype as Gfi-1^−/−^, including reduced growth and profound neutropenia (134, 135).

The constitutive neutropenia in Gfi-1-deficient mice can, to a certain extent and for a short period of time, be compensated by the adoptive transfer of neutrophils or their precursors. This approach has been used to study the role of neutrophils in the K/BxN model of antibody-mediated arthritis (136). Gfi-1^−/−^ mice were resistant to the development of arthritis in this model. Arthritis could, however, be restored, following sub-lethal irradiation and engraftment with WT bone marrow cells (a process which resulted in the production of mature neutrophils within 2 weeks) before the transfer of K/BxN serum. By contrast, arthritis was not restored upon adoptive transfer of bone marrow cells from C5aR-, CD11a-, or FcRγ-deficient mice, suggesting that expression of these molecules on neutrophils is required for the development of arthritis in this model (136).

Although Gfi-1-deficient mice are useful tools to assess the role of neutrophils *in vivo*, many additional phenotypical abnormalities due to the lack of Gfi-1 expression have to be taken into account when interpreting findings obtained with these mice. First, disrupting neutrophil differentiation leads to the appearance of an abnormal myeloid cell population, which is still capable of inducing inflammation and can act as effector cells (124, 130). Moreover, Gfi-1^−/−^ mice present with thymus aplasia (124, 130, 134), abnormal differentiation of dendritic cells (137), iNKT cells (138) and T-cells (124, 130, 134), aberrant production of IL-2, TNF, IL-10, and IL-1b (124, 133), development of eye inflammation and the presence of abscesses (124, 130). Gfi-1 was later shown to be also expressed in developing inner ear, retina, brain and dorsal root ganglia (139, 140). In accordance with these reports, Gfi-1 deficiency results in a complete loss of hair cells by apoptosis before birth as well as in a severe loss of spiral ganglion neurons that is visible 5 months after birth (140). This causes behavior defects as Gfi-1 mutant mice are ataxic, do not respond to noise, circle, and develop head tilt (140). Most importantly, Gfi-1 mutant mice exhibit delayed growth and higher mortality; these features are undoubtedly a limiting factor for long-term studies (124, 130). They are significantly restored when Gfi-1^−/−^ mice are kept in SPF conditions (141). Along this line, the generation of Gfi-1^fl/fl^ mice (133) provides a great tool for the restriction of the mutation to neutrophils and limitation of negative side effects. It was, however, reported that 10–20% of Gfi-1^fl/fl^ mice exhibited features of Gfi-1^−/−^ mice, such as reduced body size, even though no Cre was expressed (133).

### Genista Mice

An additional mouse strain was generated and was named “*Genista*” mice. In these mice, Gfi-1 activity is impaired due to a point mutation in one of the zinc finger domains following N-ethyl-N-nitrosourea-induced random mutagenesis (142, 143). The C318Y point mutation in the third zinc finger domain in the Gfi-1 gene does not affect Gfi-1 expression but is likely to affect the interaction of Gfi-1 with DNA. Strikingly, it leads to the generation of highly neutropenic mice but did not result in delayed growth or increased mortality, even in conventional non-SPF conditions (142, 143). Furthermore, although the authors reported reduced cell numbers in the thymus of *Genista* mice, and reduced B cell precursors, these mice exhibited normal T and B cell differentiation and function (142). Thus, this model of neutropenia exhibits less phenotypic abnormalities than Gfi-1^−/−^ mice (142, 143).

Nevertheless, NK cells from *Genista* mice have been shown to exhibit impaired responsiveness *in vivo* and *in vitro* (143). Indeed, neutrophils are thought to be central actors for the differentiation and activation of NK cells (143, 144) and their absence leads to impaired maturation and function of NK cells (143). Furthermore, it has also been shown that in *Genista* mice, interfering with neutrophil differentiation leads to the appearance of abnormal CD11b^+^/Ly-6G^int^ myeloid cells. Interestingly, this population appears to be arrested right after the metamyelocytic stage (142) and thus, in a more mature stage than in Gfi-1^−/−^ mice (124, 130). Mature neutrophils are thought to be necessary for autoantibody-induced arthritis (136, 145), and immune complex-mediated alveolitis (146). Surprisingly, and different to results obtained in Gfi-1^−/−^ mice, *Genista* mice still developed significant inflammation in these two models, albeit at reduced levels when compared to WT mice (142). This suggests that residual CD11b^+^/Ly-6G^int^ cells even though not fully mature are capable of sustaining inflammatory processes, and thus, question the use of *Genista* mice for *in vivo* studies of neutrophil functions. Intriguingly, however, *Genista* mice failed to provide resistance to acute bacterial infection (142), implying that neutrophil features acquired after the metamyelocytic stage are required for these effector functions. Consecutively, *Genista* mice have been used to study neutrophil functions in different models, for example in host defense against certain pathogens (67, 147, 148).

### LysM-Cre Mcl-1^fl/fl^

Myeloid cell leukemia-1 (Mcl-1) belongs to the anti-apoptotic Bcl-2 family. It is expressed by neutrophils and plays a role in delaying apoptosis, especially when neutrophils are activated (149, 150). Neutrophils rely on Mcl-1 for survival (151), as it represents the only member of the Bcl-2 family expressed by neutrophils (149). Using the Mcl-1^fl/fl^ mice expressing Cre under the LysM promoter (152), Dzhagalov et al. deleted Mcl-1 in neutrophils and macrophages (153). LysM-Cre Mcl-1^fl/fl^ mice exhibited a 3-fold decrease in blood neutrophils and a high percentage of apoptotic neutrophils as compared to WT mice, whereas the phenotype of macrophages did not seem to be affected (153). Residual neutrophils still expressed Mcl-1 and thus, had escaped Cre-mediated gene deletion, which represents a potential limitation for the use of LysM-Cre Mcl-1^fl/fl^ mice (153).

A few years later, LysM^Cre/Cre^ Mcl-1^fl/fl^ mice that are homozygous for the Cre gene were described (7), as opposed to the previously described LysM-Cre Mcl-1^fl/fl^ mice (153). This bi-allelic expression of the Cre-recombinase resulted in severely neutropenic mice with a reduction of over 93% in the spleen and bone marrow, and a 98% reduction of circulating neutrophils in homeostatic conditions (7), and which remained neutropenic even upon thioglycolate-induced peritonitis (7). None of the other cell populations assessed—circulating monocytes, eosinophils, B and T cells, bone marrow dendritic cells and macrophages, splenic dendritic cells, T and B cells—seems to be affected by this mutation, with the exception of a decrease in bone marrow B cells and an increase in splenic macrophages (7). LysM^Cre/Cre^ Mcl-1^fl/fl^ mice can breed as homozygous but display higher mortality rates and reduced offspring numbers in SPF and non-SPF conditions (7). LysM^Cre/Cre^ Mcl-1^fl/fl^ mice were used to emphasize the fact that neutrophils are necessary for the sensitization and elicitation phases of 2,4,6-trinitrochlorobenzene-induced contact hypersensitivity (154), and were, as expected, protected against K/BxN serum-transfer arthritis and anti-CVII antibody–induced dermatitis, while displaying an increased susceptibility to infection with *S. aureus* and *C. albicans* (7). When interpreting results obtained with these mice, one should bear in mind that these mice are also knockout for Lysozyme M in all cells.

### MRP8-Cre Mcl-1^fl/fl^

The same authors also crossed Mcl-1^fl/fl^ mice with mice expressing Cre under the control hMRP8 promoter (7). Indeed, MRP8-Cre Mcl-1^fl/fl^ mice showed severe neutropenia (with more than 99% reduction in circulating neutrophils as compared to WT mice, whereas blood monocytes, eosinophils, T and B cells were not affected by the mutation (7). Unfortunately, this severe neutropenia was accompanied by high mortality (only 30% survival at 1 year of age) and poor breeding productivity (7), hampering the use of these mice for *in vivo* studies.

### Foxo3a:Foxo3a-Deficient Mice

The forkhead class O (FOXO) subfamily of transcription factors are key in regulating the apoptosis, proliferation and control of oxidative stress in immune cells (155). Foxo3a is the main FOXO member expressed in lymphoid organs, and Foxo3a^−/−^ mice (156) were shown to exhibit exaggerated lymphoproliferation and inflammatory reactions in various organs (156). This phenotype was accompanied with the presence of over-activated T helper cells releasing high amounts of Th1 and Th2 cytokines (156). It was further shown that Foxo3a was an inhibitor of the NF-κB pathway, and that its absence unleashed pro-inflammatory gene signature in Foxo3a^−/−^ mice (156). With respect to its action in neutrophils, it was shown that Foxo3a^−/−^ mice were resistant to neutrophil-dependent inflammatory reactions, such as immune complex-mediated arthritis, or thioglycollate-induced peritonitis (157). Indeed, Foxo3a-deficient neutrophils were more susceptible to Fas ligand-induced apoptosis (157) which engendered an incapacity of neutrophils to persist in inflamed tissues and subsequently a neutropenia (157). This suggests Foxo3a^−/−^ mice could be used as a model to assess the function of neutrophils during inflammation, although the inflammatory state of these mice as well as the high numbers of apoptotic neutrophils could be limitations to the use of these mice.

The characteristics of genetic models of inducible and constitutive neutropenia are presented in Table 2.

**Table 2:** Characteristics of genetic models of inducible and constitutive neutropenia.

	**Mice**	**Neutrophil numbers**	**Advantages**	**Potential limitations**	**References**
Inducible	PMN^DTR^ (hMRP8-Cre iDTR^fl^)	▪ Marked reduction (>90%) in blood, spleen, bone marrow and lung neutrophils upon DT injection	▪ Normal number of neutrophils until injection of DT ▪ Neutrophil population can be restored in DT-treated PMN^DTR^ mice upon adoptive transfer of DTR^−^ neutrophils	▪ DT injection reduces levels of blood and spleen monocytes ▪ Depletion is transient and needs repetitive injections for long-term experiments ▪ Possible off-target or other side effects of repeated treatment with DT	(6)
Constitutive	CXCR2^−/−^	▪ Reduction of 60% of blood neutrophils ▪ No reduction of spleen neutrophils and higher counts of bone marrow mature neutrophils	▪ Neutrophil population can be restored upon adoptive transfer of WT neutrophils ▪ Depletion effective from birth and does not require any treatment	▪ High numbers of residual neutrophils ▪ Also expressed by other cell populations (such as monocytes/macrophages, mast cells, endothelial and epithelial cells) ▪ Side effects caused by CXCR2 deficiency such as neutrophil-independent exaggeration of inflammatory reactions	(113)
	G-CSFR^−/−^	▪ Reduction of 80 and 50% of blood and bone marrow neutrophils	▪ Neutrophil population can be restored upon adoptive transfer of WT neutrophils ▪ Depletion effective from birth and does not require any treatment	▪ High numbers of residual neutrophils ▪ Side effects caused by G-CSFR deficiency ▪ Potential appearance of compensatory mechanisms due to constitutive neutrophil deficiency	(104)
	Gfi-1^−/−^	▪ Quasi absence of mature neutrophil numbers in the blood, spleen and bone marrow	▪ Neutrophil population can be restored upon adoptive transfer of WT neutrophils ▪ Depletion effective from birth and does not require any treatment	▪ Emergence of an abnormal myeloid cell population that can induce inflammation reactions ▪ Thymus aplasia ▪ Abnormal differentiation of immune cell populations (such as dendritic cells, iNKT cells and T-cells) ▪ Potential appearance of compensatory mechanisms due to constitutive neutrophil deficiency ▪ Aberrant production of cytokines ▪ Manifestation of eye inflammation and abscesses ▪ Behavior abnormalities (ataxia, no response to noise and head tilt) ▪ Delayed growth and high mortality	(124, 130)
	Ella-Cre Gfi-1^fl/fl^	▪ NA	▪ Neutrophil population can be restored upon adoptive transfer of WT neutrophils ▪ Depletion effective from birth and does not require any treatment ▪ Gfi-1^fl/fl^ mice could be used to restrict the mutation to neutrophils	▪ Emergence of an abnormal myeloid cell population that can induce inflammation reactions ▪ Delayed growth and high mortality of Ella-Cre^+^; Gfi-1^fl/fl^ mice ▪ 10–20% of Gfi-1^fl/fl^ mice exhibited features of Gfi-1^−/−^ mice, such as reduced body size, even with no Cre expression ▪ Thymus aplasia ▪ Potential appearance of compensatory mechanisms due to constitutive neutrophil deficiency	(131, 133)
	Gfi-1^GFP/GFP^	▪ Quasi absence of mature neutrophil numbers in the blood and bone marrow	▪ Neutrophil population can be restored upon adoptive transfer of WT neutrophils ▪ Depletion effective from birth and does not require any treatment ▪ Gfi-1 expressing cells are GFP^+^ and allow the study of Gfi-1 expressing cells	▪ Emergence of an abnormal myeloid cell population that is capable of inducing inflammation reactions ▪ Thymus aplasia ▪ Abnormal differentiation of immune cell populations (such as T-cells) ▪ Potential appearance of compensatory mechanisms due to constitutive neutrophil deficiency ▪ Delayed growth and high mortality	(134, 135)
	Genista (Gfi-1^C318Y^)	▪ Marked reduction (around 90%) in blood neutrophils	▪ Normal growth and mortality, even in conventional non-SPF conditions ▪ Normal T and B cell differentiation and function ▪ Neutrophil population can be restored upon adoptive transfer of WT neutrophils ▪ Depletion effective from birth and does not require any treatment	▪ Impaired responsiveness of NK cells ▪ Emergence of abnormal CD11b^+^/Ly-6Gint myeloid cells capable of sustaining inflammatory processes ▪ Thymus aplasia ▪ Potential appearance of compensatory mechanisms due to constitutive neutrophil deficiency	(142, 143)
	LysM-Cre Mcl-1^fl/fl^	▪ 3-fold decrease in circulating neutrophils ▪ High percentage of apoptotic neutrophils	▪ Neutrophil population can be restored upon adoptive transfer of WT neutrophils ▪ Depletion effective from birth and does not require any treatment	▪ High numbers of residual neutrophils escaping deletion ▪ Knock-out of Lysozyme M ▪ Appearance of compensatory mechanisms due to constitutive neutrophil deficiency	(153)
	LysM^Cre/Cre-^ Mcl-1^fl/fl^	▪ Marked reduction of neutrophil counts in the blood (98%), spleen and bone marrow (>93%).	▪ Most of the other immune cell populations seem unaffected by the mutation ▪ Neutrophil population can be restored upon adoptive transfer of WT neutrophils ▪ Depletion effective from birth and does not require any treatment	▪ Decrease in bone marrow B cells and increase in splenic macrophages ▪ Higher mortality rate and reduced offspring numbers in SPF and non-SPF conditions ▪ Knock-out of Lysozyme M ▪ Potential appearance of compensatory mechanisms due to constitutive neutrophil deficiency	(7)
	hMRP8-Cre Mcl-1^fl/fl^	▪ Marked reduction (>99%) in blood neutrophil counts	▪ Other immune cell populations seem unaffected by the mutation ▪ Neutrophil population can be restored upon adoptive transfer of WT neutrophils ▪ Depletion effective from birth and does not require any treatment	▪ High mortality (only 30% survival at 1 year of age) ▪ Low breeding productivity ▪ Appearance of compensatory mechanisms due to constitutive neutrophil deficiency	(7)
	Foxo3a ^−/−^	▪ 50% reduction of circulating neutrophils	▪ Neutrophil population can be restored upon adoptive transfer of WT neutrophils ▪ Depletion effective from birth and does not require any treatment	▪ High numbers of apoptotic neutrophils ▪ Exaggerated lymphoproliferation and more prone to inflammation reactions ▪ Overactivated T-helper cells	(157)

## Knockout of Key Neutrophil Mediators

Neutrophils exert a multitude of mechanisms of action that are based on different key enzymes. Knockout models of these enzymes have been developed in the last decades and allowed the study of their precise roles. The main models are summarized in Table 3, as well as their limitations that need to be kept in mind when using these mice.

**Table 3:** Characteristics of models of knockouts of neutrophil key enzymes.

**Mutant**	**Gene name**	**Phenotype or limitations**	**References**	**Pharmacological inhibitor**
MPO^−/−^	Myeloperoxidase	▪ Increased ROS production by neutrophils ▪ Impaired PMA-induced NETosis ▪ Normal neutrophil counts in diverse tissues tested ▪ Expressed by other cell types (such as monocytes, macrophages, peritoneal B lymphocytes)	(158–162)	4-Aminobenzoichydrazide
NE^−/−^	Elane	▪ Impaired neutrophil functions (NETosis, pro-inflammatory cytokines release, phagocytosis and transmigration) ▪ Normal circulating leukocyte counts	(159, 163–165)	Sivelestat
Cathepsin G^−/−^	Cathepsin G	▪ Decreased production of mast cell protease-2. ▪ Normal hematopoietic population counts in diverse tissues tested ▪ Expressed by other cell types (such as splenic dendritic cells and microglia).	(166)	β-keto-phosphonic acid
PAD4^−/−^	Protein arginine deiminase 4	▪ Increased bone marrow hematopoietic multipotent progenitor numbers ▪ Normal neutrophil morphology and differentiation ▪ Normal circulating neutrophil counts ▪ Expressed by other cell types (such as circulating eosinophils and monocytes) ▪ Impaired NETosis	(167–170)	Cl-Amidine
Ncf1^*/*^	Neutrophil cytosolic factor 1	▪ Development of spontaneous chronic severe arthritis postpartum ▪ Impaired ROS production by neutrophils and macrophages ▪ Disrupted T cell functions ▪ Impaired NETosis	(171–175)	
Proteinase 3^−/−^	Proteinase 3	▪ Increased bone marrow hematopoietic stem progenitor numbers	(176)	
Lactoferrin^−/−^	Lactoferrin	▪ Normal neutrophil morphology and differentiation ▪ Normal neutrophil functions (phagocytosis, granule mobilization) although defects in PMA-induced oxidative burst ▪ Decrease in circulating lymphocyte counts but increase in circulating neutrophil and eosinophil numbers ▪ Also expressed in monocytes, macrophages and subpopulations of dendritic cells	(177–179)	

First, one has to bear in mind the abnormalities that can be induced by the knockout itself. For instance, knockout of the iron-binding protein lactoferrin (an enzyme produced by neutrophils and which has anti-microbial as well as anti-inflammatory properties) leads to a 6% decrease in circulating lymphocyte counts, but to an increase in circulating neutrophil and eosinophil numbers (of respectively 46 and 89%) (177). This could lead to phenotypic particularities that are not directly linked to a role of lactoferrin.

Neutrophil enzymes are often closely related and can exhibit redundant roles, which can limit the use of mice deficient for only one of these enzymes. For instance, neutrophil elastase (NE), cathepsin G and proteinase 3 are 3 serine proteases (180). Double mutants have therefore recently been developed to demonstrate the roles of NE and cathepsin G in the endotoxic shock cascade (181) and in lung defense against *S. pneumoniae* (182), and of NE and proteinase 3 in immune complex-induced neutrophil activation (183).

In addition, neutrophil enzymes are generally not restricted to neutrophils but are also expressed by other cell populations. This suggests that the exclusive use of enzyme-knockout models might lead to an over-estimation of the roles of neutrophils. For instance, MPO is also expressed by monocytes, macrophages and peritoneal B lymphocytes (184); and Cathepsin G by splenic dendritic cells and microglia (185). Furthermore, in addition to its role in NETosis, protein arginine deiminase 4 (PAD4) is implicated in macrophage extracellular trap release (186). To circumvent these difficulties, a conditional model of PAD4 knockout has been developed by flanking the PAD4 gene with *loxP* sequences (167). Using the neutrophil-specific promoter MRP8 (95), MRP8-Cre PAD4^fl/fl^ have been generated to restrict PAD4 deletion to neutrophils, and to demonstrate that NETs can facilitate the formation of premetastatic niches in a context of ovarian cancer (187). This approach could in principle be adapted to all neutrophil key enzymes for the generation of neutrophil-restricted knockouts, to facilitate the analysis of the real contribution of neutrophils *in vivo*.

Finally, it is noteworthy that a new mouse model has recently been generated in which the caspase recruitment domain-containing protein 9 (CARD9) is selectively knockout in neutrophils (Mrp8-Cre^+^ *Card*9^flox/flox^) (188). Mice carrying this mutation do not develop autoimmune syndromes such as autoantibody-induced arthritis or skin blistering (188), due to a lack of chemokine/cytokine release from CARD9-defient neutrophils through Src-family kinases, Syk, PLCγ2, Bcl10/Malt1, and NFκB (188). This model provides therefore a possibility to investigate the functions of neutrophil-released chemokine/cytokine in diverse physiological and pathological settings.

## *In vivo* Tracking of Neutrophils

Tracking neutrophils temporally and spatially, or assessing their activity can be informative when studying these cells *in vivo*. Indeed, it gives clear evidence of their dynamics, and it may help to dissect the cellular and molecular pathways implicated in their release from the bone marrow as well as in their recruitment to sites of inflammation.

### Assays Using *in vitro* Imaging System (IVIS)

Luminol is a chemical molecule that can directly be oxidized by HOCl produced by myeloperoxidase (MPO), or by MPO using other reactive oxygen species (ROS) (189). Oxidized luminol emits photons and can therefore be used as a mean to reveal the presence of active MPO (190). Using a *in vivo* imaging system (IVIS), it has been shown that systemic treatment of mice with luminol lead to the formation of a bioluminescent signal in tissues infiltrated by activated neutrophils in contexts of acute dermatitis, and arthritis (190, 191). No signal was generated in inflamed tissues of MPO^−/−^ mice, even if these mice showed the infiltration of activated eosinophils (190). This suggests that eosinophil peroxidase failed to induce a luminescent signal and further shows that luminol-induced signal is specific to MPO. Luminol has extensively been used as a mean to demonstrate the occurrence of inflammation reactions as well as the infiltration of neutrophils in tissues, for instance during anaphylaxis reaction (191), tumorigenesis (192) or acute and chronic intestinal inflammation (193). One caveat of luminol-based assays is the fact that neutrophils are not the exclusive producers of MPO, since this enzyme is also produced by monocytes/macrophages (184).

Other fluorescent probes have been developed following the same model, such as the Neutrophil Elastase 680 FAST imaging agent, allowing the imaging of NE activity in mice (194), as shown in a context of occurrence of colorectal cancer (195). Finally, probes binding specifically to neutrophil membrane have been generated, such as the cFIFIF-PEG-Cy7 that interacts with neutrophil formyl peptide receptor (196). This probe was for example validated in a context of ear inflammation (196).

### Neutrophil Tracking Using Intravital Microscopy

The development of imaging technologies such as intravital two- and multi-photon microscopy gave rise to the development of new strategies to track neutrophils *in vivo*. Indeed, these technologies allow the live imaging of neutrophils deep in the tissues.

First, *in vivo* tracking of neutrophils can be performed using adoptive transfers of pre-stained neutrophils. This can be performed by isolating neutrophils from the bone marrow of donor mice, and staining these cells with a fluorescent dye such as CarboxyFluorescein Succinimidyl Ester [CFSE, that is cell permeable and binds to covalently to intracellular proteins (197)] prior to transfer of the cells into recipient mice (198). Although this requires a heavy experimental procedure, it allows the use of mutant donor mice to target the molecular pathways implicated in the process of interest. This strategy was for instance employed to show that expression of the receptor IL-1R on the surface of neutrophils does not play a major role in the recruitment of these cells in inflamed tissues in a context of *Staphyloccocus aureus* infection (198).

Furthermore, labeled antibodies can be systemically injected to stain neutrophils *in vivo*. Indeed, It has been shown that low doses of anti-Ly6G antibodies do not interfere with neutrophil biology (76). No difference was thereby shown in the lung trafficking of red blood cells and neutrophils in a mouse model of Sickle cell disease when compared with WT mice (199).

To circumvent the need to treat mice with antibodies, it is possible to use or generate new strains of mice expressing reporter genes in neutrophils. For instance, LysM-EGFP mice were generated by inserting the *GFP* gene into the lysozyme M locus (152). In these mice, GFP^+^ cells are mainly neutrophils and monocytes/macrophages (152). LysM-EGFP mice were used to track neutrophils *in vivo* using two-photon intravital microscopy to show for instance that neutrophils do not directly interact with mycobacteria in the liver of infected mice (200). LysM promoter is also active in monocytes and macrophages (152) neurons (201) and alveolar type II cells in the lungs (202). This lack of cell specificity could thus potentially limit the use of LysM-EGFP mice for the imaging of neutrophils. One strategy to bypass this has been to deplete monocytes and macrophages by treating LysM-EGFP mice with clodronate liposomes to visualize the recruitment of neutrophils in atherosclerotic lesions (203). Nevertheless, the side effects of the clodronate liposome treatment need to be taken into account when analyzing the data. Another strategy could be to target a more selective promoter, such as the neutrophil-related protein Ly6G (30).

Catchup mice were generated by replacing the first exon of *Ly6g* by the “*Cre-tom*” sequence corresponding to the sequences of the Cre recombinase and the fluorescent molecule tdTomato (77). Homozygous mice exhibit endogeneous staining of neutrophils in red, but are *Ly6g* deficient. To increase the levels of the tdTomato reporter gene expression and to restore the expression of Ly6G, Catchup mice were crossed with mice expressing the fluorescent molecule tdTomato under control of the Cre-dependent promoter in the ubiquitous *ROSA26* locus (204). These *Ly6g*(Cre-tom; wt) *ROSA26*(tom; wt) mice were called Catchup^IVM−red^ mice. These mice express high levels of tdTomato in 80–90% of bone marrow, blood and spleen neutrophils, while red fluorescence appears to be restricted to neutrophils. Nevertheless, it is to note that Catchup^IVM−red^ neutrophils express slightly decreased levels of Ly6G when compared to WT neutrophils (77). Neutrophils from Catchup^IVM−red^ mice function normally *in vitro* and *in vivo*, they exhibit the same morphology as WT neutrophils and are easy to track, being endogenously stained in red. Using intravital two-photon microscopy, it was possible to show that neutrophils from Catchup^IVM−red^ mice lacking FcγRIV exhibit impaired recruitment to inflamed tissues in a context of experimental epidermolysis bullosa.

MRP8-Cre mice express the Cre recombinase followed by an IRES-GFP reporter gene. This allows detection of GFP at low levels in neutrophils by flow cytometry (6, 95). However, GFP expression is likely too low to permit detection of neutrophils by microscopy using MRP8-Cre mice. However, MRP8-Cre mice were crossed with mice expressing a Cre-inducible YFP gene under the control of the ROSA26 locus. This lead to high levels of YFP expression in ~ 80% bone marrow, blood and spleen neutrophils (6, 97). However, YFP expression was also reported in 10–20% of some monocyte and macrophages populations in MRP8-Cre YFP^fl^ mice (97), which limits the use of these mice for imaging approaches.

## *In vivo* Characterization of NETs

NETosis is an innate immune mechanism that involves the release of DNA decorated with histones and granule enzymes upon neutrophil activation (205). Since their first description in 2004 (4), NETs have extensively been studied and their contribution to human diseases has been suggested in many different settings. These include atherosclerosis (206), cancer metastasis (61), or thrombosis and allergic conditions (207–209).

Despite their reported multiple involvements, characterizing NETs *in vivo* remains challenging. Neutrophils (and other cells) can release DNA through multiple processes, including necrosis, and it is often difficult to stain DNA in-depth within the tissues and combine this staining with appropriate markers to distinguish NETs from other forms of tissular DNA. Methods to study NETosis have been reviewed elsewhere (210) and will only be briefly discussed here.

The occurrence of NETosis *in vivo* is often shown using fluorescent dyes revealing the colocalization of neutrophils (211), extracellular DNA [using non-permeable DNA intercalating agents such as Sytox Green (212), Sytox Orange (213–215) or propidium iodide (PI) (213–215)], and NET-associated molecules [i.e., NE, MPO or citrullinated histones (213–215)]. Images are then acquired by use of intravital microscopy [two-photon (206, 211, 215), multiphoton (214), or spinning disk (212, 213)]. 3D reconstructions of NETting neutrophils can even be performed to assess the morphological changes happening during NETosis (213).

## Concluding Remarks

The use of mouse models for the study of neutrophils *in vivo* represents a milestone in the understanding of the biology of these cells, and has enabled the deep interrogation of a number of pathways leading to diverse pathologies. For instance, DNase I, that digests the DNA backbone of NETs, has been approved as a therapeutic drug to treat Cystic Fibrosis after the discovery that NETs play a pivotal role in the disease (216). Whereas, early neutrophil depletion approaches often resulted in a more generalized deficiency of hematopoietic cells, recent advances in our understanding of neutrophil biology lead to the generation of more specific ways of depleting neutrophils. Nevertheless, the selectivity, specificity and the side effects of each of the different strategies need to be carefully evaluated, before concluding on neutrophil functions. The use of at least two different models seems indeed to be recommended to undoubtedly define the roles of neutrophils. Furthermore, the use of mutant mice helps to decipher the molecular pathways of interest. The expression of neutrophil key enzymes is often shared with other cell populations and the Cre-lox system will at term allow the generation of a library of mice exhibiting a deficiency of key enzymes specifically in neutrophils.

Although visualization of neutrophil activity using IVIS is an easy tool to assess the contribution of neutrophils *in vivo*, recent advances in intravital microscopy allow more precise tracking of neutrophils *in vivo*. These imaging approaches permit the observation of the behavior of the neutrophils, their interactions with other cell populations, and may finally help to answer some debated aspects of neutrophil biology. However, the analysis of deep organs still remains challenging.

Today, *in vivo* tracking of NETosis clearly remains one of the most challenging tasks. While it is relatively straightforward to visualize neutrophils and extracellular DNA, this co-staining is not sufficient to provide convincing proof of NETs. Indeed evidence for the presence of histones or signature enzymes associated to the DNA is a minimum necessity. Further difficulties result from the fact that there is an ongoing debate on the definition of NETosis, and as a consequence NETs. Indeed many different signaling pathways have been put forward and proposed to result in either suicidal, nuclear NETosis or mitochondrial vital NETosis (217). Moreover, the observation of NETs in a given pathology does not necessarily mean that NETs play a role in such disease, and proving so can be very challenging.

In conclusion, there is now a wide range of tools for assessing the functions of neutrophils *in vivo*. The main issue is therefore the choice of the methods used. Therefore, one needs to consider each advantage and caveat of these methods. Furthermore, confirming the results using at least two different approaches seems necessary to avoid overestimating or underestimating the true contribution of neutrophils *in vivo*.

## Author Contributions

JS, FJ, and LR wrote the review.

### Conflict of Interest

The authors declare that the research was conducted in the absence of any commercial or financial relationships that could be construed as a potential conflict of interest.
